# Supplementary material for: Dual-Energy Computed Tomography-Based Iodine Concentration Estimation for Evaluating Choroidal Malignant Melanoma Response to Treatment: Optimization and Primary Validation
Source: Diagnostics (Basel). 2022 Nov 4;12(11):2692. doi: 10.3390/diagnostics12112692 (PMC9689166; doi:10.3390/diagnostics12112692)
Supplement: Supplementary file 1 [file diagnostics-12-02692-s001.zip › diagnostics-1928963-supplementary.pdf]

Supplementary material

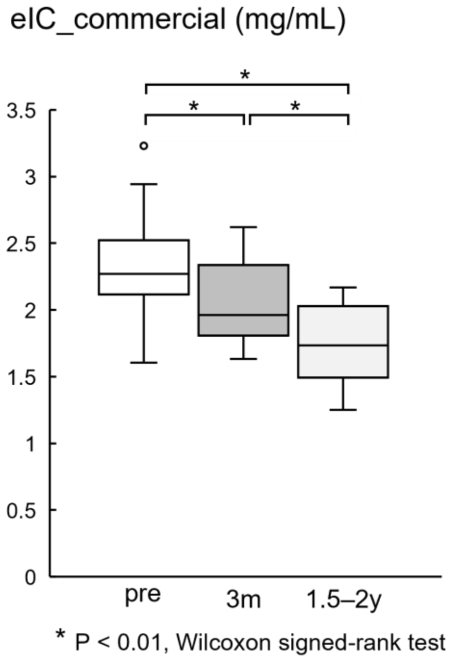

Figure S1:

The eIC_commercial decreased significantly in comparisons between the values obtained pre-treatment and 3 months after treatment, and between those obtained 3 months and 1.5-2 years after treatment.
